# Treatment Methods and Early Neurologic Improvement After Endovascular Treatment of Tandem Occlusions in Acute Ischemic Stroke

**DOI:** 10.3389/fneur.2019.00127

**Published:** 2019-02-27

**Authors:** Marta Wallocha, René Chapot, Hannes Nordmeyer, Jens Fiehler, Ralph Weber, Christian Paul Stracke

**Affiliations:** ^1^Department of Radiology and Neuroradiology, Alfried Krupp Krankenhaus Essen, Essen, Germany; ^2^Department for Neuroradiological Diagnosis and Intervention, University Medical Center Hamburg-Eppendorf, Hamburg, Germany; ^3^Department of Neurology, Alfried Krupp Krankenhaus, Essen, Germany

**Keywords:** tandem occlusion, stroke, endovascular treatment, emergency stenting, thrombectomy

## Abstract

**Background and Purpose:** A tandem occlusion of the intracranial circulation and the extracranial carotid artery (ICA) occurs in 10–20% of all strokes based on large vessel occlusion (LVO). The optimal treatment strategy for those patients is unknown. We report our management strategy and the outcome in these patients in a large single-center cohort.

**Materials and Methods:** We retrospectively identified and analyzed all patients treated by Mechanical Thrombectomy (MT) for an intracranial LVO associated with an occlusion of the extracranial ICA between April 2009 and May 2016 (163/1,645, 9.9%). The following data was collected: Recanalization rate, occurrence of symptomatic intracranial hemorrhage (sICH), clinical result according to the early neurological improvement (ENI, NIHSS score improvement of ≥8 points after 24 h or NIHSS score of 0 or 1 after 3 days) and functional outcome and mortality during long term follow up. Secondary endpoints were the patency of the internal carotid artery at 24 h. Patient demographics and anti-aggregation regimen were recorded as co-variables.

**Results:** 163/1,645 (9.9%) MT patients had a tandem occlusion. All thrombectomy procedures were performed with stent retrievers. PTA with or without additional placement of a stent was performed in 149 vs. 14 patients. The overall rate of TICI IIB/III recanalization was 91.4%. An early neurological improvement was found in 79 of 163 patients (48.4%), 51% (76/149) in the stent group and 21% (3/14) in the non stent group. 120/163 patients (73.6%) had a long term favorable outcome (mRS 0–2). The ICA re-occlusion rate at 24 h was 5.4% (8/149) in the stent group and 42% (6/14) in the non stent group. The rate of symptomatic hemorrhage was 4.9%.

The regression analysis showed that only younger age (*p* = 0.002) and shorter recanalization times (*p* = 0.017) were associated with good outcome.

**Conclusion:** Stent-PTA of the ICA in addition to MT with a stent retriever was safe and effective in tandem occlusion of the anterior brain circulation. PTA and MT without stenting in tandem lesions showed a higher early re-occlusion rate and lower rate of early neurological improvement. The technical approach should aim for the fastest possible recanalization of the intracranial vessels, either with stenting first or last.

## Introduction

In stroke patients with large vessel occlusion (LVO), mechanical thrombectomy (MT) has become the standard of care since randomized trials have proven superiority over intravenous thrombolysis alone (1–5). A complete or subtotal occlusion of the extracranial internal carotid artery (EICA) is present in 10–20% of strokes with a LVO (6). Patients with tandem occlusions of the ICA had a poor clinical outcome and treatment response to intravenous thrombolysis in previous studies (7, 8). In the randomized studies that included those patients (1, 3, 4), the amount of patients with tandem lesions remained low and the specific treatment method unclear. Therefore, no recommendations have been given for the treatment of acute tandem occlusions.

Several papers on case series with tandem occlusions reported encouraging results for the combination of MT and stenting of the EICA (9–14). Nonetheless, the small patient numbers in each case series limit the validity of the findings. A review of 32 studies showed that acute stenting of EICA in patients with tandem occlusion resulted in a significantly better outcome (15). Behme et al. (16) published a retrospective multicenter series including 170 patients supporting the combination of carotid stenting and intracranial mechanical thrombectomy even though there were variations among the centers in the periprocedural management of antiplatelet regimen.

The treatment of acute tandem occlusion raises several questions concerning the treatment of the stenosis and the required anti-aggregation regimen. The stenosis can be treated by PTA alone or in combination with stent placement. The reported antiplatelet regimens range from no anti-aggregants, single, or double medication to G2B3A antagonists. Anti-aggregation is required to prevent a thrombosis of the stent but may induce a higher rate of intracranial hemorrhage (9) in combination to intravenous lysis.

The purpose of this clinical study is to analyze the results of the endovascular treatment and periprocedural management of tandem occlusions in a single center and report the outcome related to specific endovascular methods (with/without permanent stent and proximal/distal recanalization first) and antiplatelet regimens.

## Methods

### Patients and Diagnosis

We retrospectively analyzed in our prospective database all consecutive patients with an acute atherosclerotic occlusion of the EICA and an intracranial occlusion of the anterior circulation treated by PTA or Stent/PTA and intracranial MT in our department between April 2009 and May 2016.

Since 2008, all MTs in our department were performed with stent retrievers in combination to balloon guiding catheters. No intermediate catheters or aspiration- alone techniques were used in this cohort. Dissections of the extracranial carotid artery as the cause of the EICA occlusion were excluded. There was no absolute time threshold for treatment delay from symptom onset. The diagnosis of intracranial LVO and cervical ICA occlusion was obtained by CT angiography. Early Neurologic Improvement (ENI) was assessed based on the NIH Stroke Scale (NIHSS) score assessed by a stroke Neurologist at admission and every 6 h. The modified Ranking Score (mRS) was obtained 3 months to 5 years after the procedure by a Neurologist or if not accessible, based on hospital discharge or rehabilitation letters or direct phone call.

A CT-scan with CT angiography was obtained in all patients prior to MT to assess the intracranial LVO of the anterior circulation and EICA occlusion and exclude an intracranial hemorrhage or an extensive brain infarction. Twenty four hours patency of the treated EICA was systematically assessed by duplex ultrasound by an experienced stroke neurologist. A plain CT scan was performed 24 h after MT.

### Endovascular Procedure

The procedures were done under general anesthesia. Five *Interventionalists with 21, 14, 6, 6, and 5 years of experience in endovascular procedures performed the treatments*.

**Figure 1:**
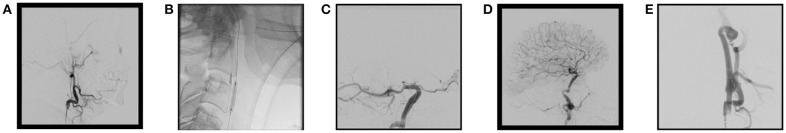
**(A)** Extracranial occlusion of the Internal Carotid Artery. **(B)** Ballon-PTA (5 mm) before passing with the 8F-Guiding Catheter. **(C)** Carotid-T-occlusion with already placed Stent retriever. **(D)** TICI III recanalization after thrombectomy. **(E)** Stent-placement in extracranial internal carotid artery after thrombectomy.

An 8F Balloon Guiding Catheter (BGC) (Concentric Medical, Mountain View, CA, USA and Flow Gate 2, Stryker, Kalamazoo, MI, United States) was navigated from the groin to the common carotid artery. Whenever possible, the 8F BGC, supported by a 125 cm 5F or 6F diagnostic catheter, was pushed through the angiographically *completely occluded* EICA after blind catheterization with a Terumo 0.035 guidewire (Terumo Europe, Leuven, Belgium). The intracranial LVO was then treated by MT followed by PTA or PTA/Stenting of the EICA (retrograde approach). Whenever access to the internal carotid artery could not be obtained with the 8F BGC, a PTA, or stent PTA was performed first (antegrade approach) (Figure 1).

Distal recanalization was systematically achieved with a stent retriever, mostly the Solitaire (Medtronic, Dublin, Ireland). The stent-retriever was retrieved under continuous manual aspiration after inflation of the balloon of the BGC. The maneuvers were repeated with a single device up to 3 times. In case of persistent failure, MT was achieved by simultaneous retrieval of 2 stent retrievers deployed in parallel.

Proximal recanalization was achieved with a 5 or 5.5 mm PTA Balloon (Falcon, Invatec S.p.A., Rocadelle, Italy; Ultrasoft, Boston Scientific, Maple Grove, Minnesota, USA). Stenting with a 7 × 40 carotid Wallstent (Boston Scientific, Natick, MA, USA) was either achieved before PTA in the retrograde approach or after PTA in the antegrade approach. In some patients cases, a PTA alone was done without implantation of a stent (No Stent group). No distal protection devices or filter systems were used during PTA or stenting. Five hundred milligram Aspirin IV was injected in the stent group, no anti-aggregation was given in the PTA alone group. *Intravenous heparinization was not used in all procedures*. Clopidogrel was started 24 h later in all stented patients when the infarction was smaller than 1/3 of the MCA territory. *Double antiplatelet therapy was continued four weeks after the procedure followed by permanent monoantiaggregation*.

### Data Analysis

The Thrombolysis in Cerebral infarction (TICI) score was assessed on digital subtraction angiography at the end of the procedure, the recanalization time (defined as time from first series to recanalization of the intracranial vessels) and the rate of symptomatic intracranial hemorrhage (sICH) on the 24 h CT scan were assessed by 2 experienced Neuroradiologists (HN and PS). The primary clinical endpoint was determined according to the definition of ENI (17, 18). ENI was considered positive in case of a reduction in the NIHSS score of ≥8 points after 24 h or a residual NIHSS score of 0 or 1 after 3 days.

Long-term outcome using the modified Rankin Scale score was dichotomized into favorable outcome (mRS 0–2) and poor outcome (mRS 3–6). Patients lost to follow-up were considered as poor outcome. To determine which independent variables were associated with a favorable outcome, we performed a logistic regression analysis with the variables sex, age, time window, intravenous tPA therapy (IVT), NIHSS at admission, recanalization time, ante- or retrograde approach and stenting duration. A Chi-Square test and a Fisher's exact test analyzed continuous variables as recanalization time, duration of stenting, age, IV Lysis, and treatment strategy (antegrade vs. retrograde approach and Stent vs. Non Stent group). Variables of strategy are presented as means (±SD) or medians. A *p*-value of 0.05 was considered as significant. All statistical analyses were performed with SPSS (IBM Armonk, NY, USA). Patients were analyzed concerning the antiplatelet therapy (Aspirin, Clopidogrel or Eptifibatide).

## Results

### Endovascular Procedure

We identified 163/1645 (9.9%) patients with a tandem occlusion that were treated by MT in our department between April 2009 and May 2016. The characteristics are summarized in Table 1. The mean age was 67.6 years (range 42–91 years). There was a male predominance with 118 male (72.4%) and 45 female patients (27.6%). The mean NIHSS was 14.6 with a range of 0–30. The mean delay from symptom onset until treatment was 234 min; 24 patients had a wake up stroke. Prior intravenous tPa was given in 90/163 patients (55.2%).

**Table 1:** Baseline, treatment and outcome variables in the PTA only and stent group.

	**PTA (*n* = 14)**	**Stent-PTA (*n* = 149)**
**BASELINE VARIABLES**		
Median Age ± SD—year	69 (±9.7)	69 (±10.7)
Male gender (%)	42.9	75.2
Female gender (%)	57.1	24.8
Median Time Window ± SD—minutes	220 (±75.1)	198 (±82)
i.v. Lysis (%)	64.3	54.4
NIHSS at admission ± SD—points	15 (±6.8)	14 (±6.0)
**PROCEDURAL CHARACTERISTICS**		
Recanalization time ± SD—minutes	39 (±36.7)	28 (±23.5)
Retrograde approach (%)	28.6	49.7
Antegrade approach (%)	64.3	40.3
extra-/intra-/extracranial (%)	7.1	10.1
Passes ± SD—number	2 (±1.7)	2 (±1.1)
Recanalization rate TICI IIb/IIIa (%)	85.7	92
Stenting duration ± SD—minutes	—	10 (±10.1)
**ANTIAGGREGATION AFTER 24 H CT- SCAN**		
ASS monotherapy	92.9	32.2
ASS + Clopidogrel	0	63.1
Phenprocoumon	0	0.7
No anti-aggregation (%)	7.1	3.3
**OUTCOMES**		
Early neurological improvement (%)	21	51
SICH (%)	7.1	4.7
Stent/ICA re-occlusion (%)	42.8	5.6
NIHSS at discharge ± SD—points	10 (±5.9)	4 (±5.8)
mRS 0–2 long term (%)	57.1	75.2
mRS 3–5 long term (%)	14.3	9.4
Death long term (%)	28.6	12.1

A TICI IIB/III recanalization was achieved in 149/163 patients (91.4%) after an average of 1.9 passes. The mean groin to recanalization time was 34 min (range 7–155 min). The sequence of treatment was a retrograde in 78/163 (47.9%) patients, and an antegrade approach in 69/163 (42.3%) patients. 149 patients had permanent stenting and 14 patients PTA only. In addition to the systematic administration of intravenous aspirin, few patients received additional medication in the acute setting including eptifibatide (*n* = 8), heparin (*n* = 1), or clopidogrel (*n* = 9).

### Imaging Outcome

6/14 (42.8%) of the carotid lesions treated with PTA only were re-occluded after 24 h proven by Doppler ultrasound. We observed a re-occlusion of the extracranial ICA- Stent within the first 24 h in 8/149 patients (5.4%), in two cases the re-occlusion was symptomatic.

Symptomatic intracranial hemorrhage (sICH), i.e., an intracranial hemorrhage associated with a deterioration of ≥4 NIHSS points, was observed in 8 patients (4.9%), one patient (7%) in the PTA group, and 7/149 (4.7%) in the stent group.

### Clinical Outcome

Early neurological evaluation could be performed in 152/163 patients. Eleven Patients could not be evaluated for following reasons: Intubation in the intensive care unit for more than 24 h (*n* = 8), early transfer to referring stroke unit (*n* = 3). Early neurologic improvement was documented in 79 patients out of 160 (49.4%) patients, 76/149 (51%) patients in the stent group and 3/11(27.2%) patients in the PTA only group. Symptomatic intracranial hemorrhage (sICH), was observed in 8 patients (4.9%), one patient (7%) in the PTA only group, and 7/149 (4.7%) in the stent group.

In the long-term follow-up, 120/163 patients (73.6%) showed a favorable outcome (mRS 0–2), 16 (10%) patients showed an unfavorable outcome (mRS 3–5) and 22 patients (13.4%) were dead (28.6%). Five patients were lost to follow-up (3%) and were imputed with unfavorable outcome. In the stent group, 112/149 patients had a good outcome (75%) and 8 patients in the PTA only group (57%).

The regression analysis showed that younger age (*p* = 0.002) and shorter recanalization times (*p* = 0.017) were associated with favorable outcome (Table 2). Ninety percentage of patients with a procedure time of < 20 min (*n* = 40) hat a good outcome (mRS 0–2). In procedures ranging from 20 to 60 min (*n* = 85), the outcome was good in 79% of patients. In procedures lasting more than 60 min (*n* = 18), only 50% of the patients had a mRS 0–2 at long-term follow-up. There was a correlation between longer recanalization time and poor outcome in patients who were additionally treated with IV tPa (*p* = 0.019) whereas there was no such association in the patients treated without tPa (*p* = 0.605). Additional iv tPa had no influence on patient outcome compared to MT alone. The median recanalization time was 6 min longer in the antegrade than in the retrograde approach. However, no significant difference in outcome was found in the retrograde vs. antegrade approach. The difference in favorable outcome in the stent/no stent groups (75% vs. 57%) was not statistically significant.

**Table 2:** Regression analysis for favorable long term outcome (mRS0–2).

**Variable**	***P*-Value**
Sex	0.477
**Age**	**0.002**
Time window	0.214
i.v. Lysis	0.979
NIHSS at admission	0.182
**Recanalization time**	**0.017**
Retrograde approach	0.777
Stenting duration	0.653

*The bold values are statistically significant*.

## Discussion

Our analysis of a large cohort of 163 patients with tandem lesions is relevant, as there is no other study in this patient subgroup with all patients being treated in the same center. This type of study guarantees relatively consistent interventional experience and standardized endovascular approaches as well as low variability after care regimens.

This retrospective analysis showed a high level of recanalization (91.4% TICI IIb and III) and a high rate of favorable early neurologic outcome with 51% of early neurological improvement in tandem occlusions treated by the combination of Stent-PTA, MT with a stent retriever and monoantiaggregation with aspirin. The actual rate of good outcome may even be underestimated as non-evaluable patients were considered to have poor outcome.

Both ENI and long term mRS showed a high rate of favorable outcome (ENI 49%, 76% mRS 0–2).

The long-term clinical follow-up was available in 97% of the patients. Our data cannot be compared with the mRS scores obtained from other studies as the interval between the initial stroke and the assessment of the mRS was variable in our study, ranging from 3 months to 5 years. However, recent analyses showed limited differences of the mRS at 90 days and the mRS at 1 or 2 years (19, 20). We do not expect that a longer-term observation intervals will increase the rate of good outcomes. Moreover, a long-term observation interval in an elderly population will also include deterioration unrelated to the initial stroke.

In order to validate our mRS data, we additionally evaluated ENI. Previous studies (17, 18, 21) have already pointed out that the ENI after 24 h or 3 days is an accurate tool to assess long term clinical outcome after a stroke. It may even be preferred to the 3 months mRS, as in Extend IA (2), where the ENI was a coprimary outcome and the 90 days mRS score only a secondary outcome parameter.

The good results could be explained by specific characteristics of tandem occlusions. The 91% recanalization rate achieved in our patients with tandem occlusions was higher than the 74% rate that was reported in our hospital in a retrospective multicenter study of 210 consecutive patients with an anterior stroke from June 2012 to August 2013 with similar MT technique (22). The higher recanalization rate may be due to the nature of the thrombus which may be easier to extract. This is supported by the lower number of passes (1.9 vs. 2.3) in tandem occlusion patients compared to the aforementioned study. A difference in the nature of the clot has also been investigated in several studies looking after a possible association between the histopathologic characteristics and mechanical properties (23, 24).

This feature even might lead to a shorter recanalization time, which in our analysis, in addition to age, was significantly associated with a better outcome, similar to recently published papers (25, 26). Another potential explanation for a better clinical outcome may be the development of collaterals in chronic stenosis of the extracranial ICA (27).

There were only 14 patients in the non-stent group as we rapidly observed a lower rate of early neurological improvement at 24 h (21%) and a higher early re-occlusion rate of the ICA (42%). These observations lead us to abandon this approach, resulting in a bias. As a consequence, the PTA only group was small and these patients did not receive anti-aggregation treatment with aspirin in contrast to the stent group.

There was no difference in clinical outcome when comparing the antegrade with the retrograde approach. Our initial hypothesis was a better outcome in the retrograde approach as patients would mostly benefit from the earliest possible intracranial recanalization. Our assumption was that the acute symptoms may rather be related to the MCA occlusion than to the EICA occlusion in this population with a chronic high grade ICA stenosis. Furthermore, successful thrombectomy allows prescription of anti-aggregants whereas the need for stent placement may be questionable in case of failure of thrombectomy. However, no difference was found between both groups, presumably because there was no significant difference in the time to achieve intracranial recanalization between both groups. This may be due to a too small sample size, but also to the short time required for PTA/stenting.

Ninety patients (55%) received IVT which had no impact referring to symptomatic intracranial hemorrhage or long term outcome, which was in agreement with recent studies (1, 3, 5). In our cohort, secondary referred patients had a higher rate of IVT and a longer time window. Due to this bias, we cannot give a reliable statement on the actual effect of IVT.

Symptomatic intracranial hemorrhage (sICH) occurred in 4.9% of all tandem occlusions in our study. This rate is comparable to the rate of sICH in the large randomized thrombectomy studies. These data suggest that monoantiagregation with aspirin does not seem to increase the bleeding rate. The 9% rate of sICH reported by Behme et al. (16) is higher than our observation. This may be explained by a double antiplatelet regimen, which is the gold standard in non-emergency stent placement, which has potentially been given in their series. The low rate of early re-occlusion (5.4%) supports the use of monoantiagregation. Furthermore, these re-occlusions were mostly asymptomatic.

There was no difference between patients treated with IVT prior to thrombectomy regarding sICH, rate in our study. Thus, aspirin monotherapy seems to be adequate to prevent stent thrombosis.

## Limitations

The main limitation of our study is its retrospective design. Due to the variable and partially very long time period for the assessment of mRS score, the reliability of the outcome data might be biased, as discussed above.

As already stated, the non-stent group was small but the poor results in this group led us to rapidly change our treatment strategy over time.

## Conclusion

We present a management strategy in the endovascular treatment of acute tandem occlusions which has shown to be safe and is associated with a good clinical outcome, based on our retrospective analysis of a large single-center cohort.

PTA and thrombectomy without stenting in tandem lesions leads to a high early re-occlusion rate. MT with a stent retriever and Stent-PTA of the ICA seems to be safe and very effective under periprocedural monoantiaggregation. The rate of sICH and stent re-occlusions seem to be acceptable in the light of very high recanalization rates and high rate of early neurological improvement and a very good clinical long-term outcome. The technical approach should aim the fastest possible recanalization of the intracranial vessels, either with stenting first or last.

## Ethics Statement

This analysis has a retrospective design and is in accordance with the Declaration of Helsinki and the responsible local ethics committee.

## Author Contributions

MW completed the data collection, analyzed the data, and wrote the paper. RC was involved mainly in the endovascular procedure and the decision about antiplatelet regimen. JF performed the statistic analysis of the data. HN was involved mainly in the endovascular procedures and analyzed the angiography data. RW performed the clinical stroke assessment and ENI analysis. CS analyzed the data of the endovascular procedure and was mainly involved in the endovascular procedure and the antiplatelet therapy.

### Conflict of Interest Statement

The authors declare that the research was conducted in the absence of any commercial or financial relationships that could be construed as a potential conflict of interest.

## References

[1] Berkhemer OA Fransen PS Beumer D van den Berg LA Lingsma HF Yoo AJ. A randomized trial of intraarterial treatment for acute ischemic stroke. N Engl J Med. (2015) 372:11–20. 10.1056/NEJMoa1411587 25517348

[2] Campbell BC Mitchell PJ Kleinig TJ Dewey HM Churilov L Yassi N. Endovascular therapy for ischemic stroke with perfusion-imaging selection. N Engl J Med. (2015) 372:1009–18. 10.1056/NEJMoa1414792 25671797

[3] Goyal M Demchuk AM Menon BK Eesa M Rempel JL Thornton J. Randomized assessment of rapid endovascular treatment of ischemic stroke. N Engl J Med. (2015) 372:1019–30. 10.1056/NEJMoa1414905 25671798

[4] Jovin TG Chamorro A Cobo E de Miquel MA Molina CA Rovira A. Thrombectomy within 8 hours after symptom onset in ischemic stroke. N Engl J Med. (2015) 372:2296–306. 10.1056/NEJMoa1503780 25882510

[5] Saver JL Goyal M Bonafe A Diener HC Levy EI Pereira VM. Stent-retriever thrombectomy after intravenous t-PA vs. t-PA alone in stroke. N Engl J Med. (2015) 372:2285–95. 10.1056/NEJMoa1415061 25882376

[6] Grau AJ Weimar C Buggle F Heinrich A Goertler M Neumaier S. Risk factors, outcome, and treatment in subtypes of ischemic stroke: the German stroke data bank. Stroke (2001) 32:2559–66. 10.1161/hs1101.098524 11692017

[7] Linfante I Llinas RH Selim M Chaves C Kumar S Parker RA. Clinical and vascular outcome in internal carotid artery versus middle cerebral artery occlusions after intravenous tissue plasminogen activator. Stroke (2002) 33:2066–71. 10.1161/01.STR.0000021001.18101.A5 12154264

[8] Rubiera M Ribo M Delgado-Mederos R Santamarina E Delgado P Montaner J. Tandem internal carotid artery/middle cerebral artery occlusion: an independent predictor of poor outcome after systemic thrombolysis. Stroke (2006) 37:2301–5. 10.1161/01.STR.0000237070.80133.1d 16888266

[9] Heck DV Brown MD. Carotid stenting and intracranial thrombectomy for treatment of acute stroke due to tandem occlusions with aggressive antiplatelet therapy may be associated with a high incidence of intracranial hemorrhage. J Neurointerv Surg. (2015) 7:170–5. 10.1136/neurintsurg-2014-011224 25387730

[10] Lockau H Liebig T Henning T Neuschmelting V Stetefeld H Kabbasch C. Mechanical thrombectomy in tandem occlusion: procedural considerations and clinical results. Neuroradiology (2015) 57:589–98. 10.1007/s00234-014-1465-5 25404414

[11] Malik AM Vora NA Lin R Zaidi SF Aleu A Jankowitz BT. Endovascular treatment of tandem extracranial/intracranial anterior circulation occlusions: preliminary single-center experience. Stroke (2011) 42:1653–7. 10.1161/STROKEAHA.110.595520 21512175

[12] Maurer CJ Joachimski F Berlis A. Two in one: endovascular treatment of acute tandem occlusions in the anterior circulation. Clin Neuroradiol. (2015) 25:397–402. 10.1007/s00062-014-0318-2 24988990

[13] Mpotsaris A Bussmeyer M Buchner H Weber W. Clinical outcome of neurointerventional emergency treatment of extra- or intracranial tandem occlusions in acute major stroke: antegrade approach with wallstent and solitaire stent retriever. Clin Neuroradiol. (2013) 23:207–15. 10.1007/s00062-013-0197-y 23354342

[14] Stampfl S Ringleb PA Mohlenbruch M Hametner C Herweh C Pham M. Emergency cervical internal carotid artery stenting in combination with intracranial thrombectomy in acute stroke. Am J Neuroradiol. (2014) 35:741–6. 10.3174/ajnr.A3763 24157733 PMC7965815

[15] Kappelhof M Marquering HA Berkhemer OA Majoie CB. Intra-arterial treatment of patients with acute ischemic stroke and internal carotid artery occlusion: a literature review. J Neurointerv Surg. (2015) 7:8–15. 10.1136/neurintsurg-2013-011004 24385555

[16] Behme D Mpotsaris A Zeyen P Psychogios MN Kowoll A Maurer CJ. Emergency stenting of the extracranial internal carotid artery in combination with anterior circulation thrombectomy in acute ischemic stroke: a retrospective multicenter study. Am J Neuroradiol. (2015) 36:2340–5. 10.3174/ajnr.A4459 26294652 PMC7964286

[17] Banks JL Marotta CA. Outcomes validity and reliability of the modified Rankin scale: implications for stroke clinical trials: a literature review and synthesis. Stroke (2007) 38:1091–6. 10.1161/01.STR.0000258355.23810.c6 17272767

[18] Kharitonova T Mikulik R Roine RO Soinne L Ahmed N Wahlgren N. Association of early National Institutes of Health Stroke Scale improvement with vessel recanalization and functional outcome after intravenous thrombolysis in ischemic stroke. Stroke (2011) 42:1638–43. 10.1161/STROKEAHA.110.606194 21512176

[19] Davalos A Cobo E Molina CA Chamorro A de Miquel MA Roman LS. Safety and efficacy of thrombectomy in acute ischaemic stroke (REVASCAT): 1-year follow-up of a randomised open-label trial. Lancet Neurol. (2017) 16:369–76. 10.1016/S1474-4422(17)30047-9 28318984

[20] van den Berg LA Dijkgraaf MG Berkhemer OA Fransen PS Beumer D Lingsma H Two-year clinical follow-up of the Multicenter Randomized Clinical Trial of Endovascular Treatment for Acute Ischemic Stroke in The Netherlands (MR CLEAN): design and statistical analysis plan of the extended follow-up study. Trials (2016) 17:555 10.1186/s13063-016-1669-6 27876083 PMC5120535

[21] Saposnik G Di Legge S Webster F Hachinski V. Predictors of major neurologic improvement after thrombolysis in acute stroke. Neurology (2005) 65:1169–74. 10.1212/01.wnl.0000180687.75907.4b 16247041

[22] Weber R Reimann G Weimar C Winkler A Berger K Nordmeyer H. Outcome and periprocedural time management in referred versus directly admitted stroke patients treated with thrombectomy. Ther Adv Neurol Disord. (2016) 9:79–84. 10.1177/1756285615617081 27006695 PMC4784249

[23] De Meyer SF Andersson T Baxter B Bendszus M Brouwer P Brinjikji W Analyses of thrombi in acute ischemic stroke: a consensus statement on current knowledge and future directions. Int J Stroke (2017) 12:606–14. 10.1177/1747493017709671 28534706

[24] Sporns PB Hanning U Schwindt W Velasco A Minnerup J Zoubi T. Ischemic stroke: what does the histological composition tell us about the origin of the thrombus? Stroke (2017) 48:2206–10. 10.1161/STROKEAHA.117.016590 28626055

[25] Ribo M Molina CA Cobo E Cerda N Tomasello A Quesada H. Association between time to reperfusion and outcome is primarily driven by the time from imaging to reperfusion. Stroke (2016) 47:999–1004. 10.1161/STROKEAHA.115.011721 26956258

[26] Saver JL Goyal M van der Lugt A Menon BK Majoie CB Dippel DW. Time to treatment with endovascular thrombectomy and outcomes from ischemic stroke: a meta-analysis. JAMA (2016) 316:1279–88. 10.1001/jama.2016.13647 27673305

[27] Romero JR Pikula A Nguyen TN Nien YL Norbash A Babikian VL. Cerebral collateral circulation in carotid artery disease. Curr Cardiol Rev. (2009) 5:279–88. 10.2174/157340309789317887 21037845 PMC2842960

